# Association between medical home characteristics and staff professional experiences in pediatric practices

**DOI:** 10.1186/2049-3258-72-36

**Published:** 2014-10-20

**Authors:** Caprice Knapp, Shourjo Chakravorty, Vanessa Madden, Jacqueline Baron-Lee, Ruth Gubernick, Steven Kairys, Cristina Pelaez-Velez, Lee M Sanders, Lindsay Thompson

**Affiliations:** Department of Health Outcomes and Policy, University of Florida, 1329 SW 16th St, Gainesville, FL 32608 USA; RSG Consulting, Cherry Hill, NJ USA; School of Public Health, University of Medicine and Dentistry of New Jersey, Newark, NJ USA; Department of Pediatrics, University of South Florida, Tampa, FL USA; Center for Health Policy, Stanford University, Stanford, CA USA; Department of Pediatrics, University of Florida, Gainesville, FL USA

**Keywords:** Medical home, Pediatrics, CHIPRA, Staff, Survey

## Abstract

**Background:**

The patient-centered medical home (PCMH) model has been touted as a potential way to improve primary care. As more PCMH projects are undertaken it is critical to understand professional experiences as staff are key in implementing and maintaining the necessary changes. A paucity of information on staff experiences is available, and our study aims to fill that critical gap in the literature.

**Methods:**

Eligible pediatric practices were invited to participate in the Florida Pediatric Medical Home Demonstration Project out which 20 practices were selected. Eligibility criteria included a minimum of 100 children with special health care needs and participation in Medicaid, a Medicaid health plan, or Florida KidCare. Survey data were collected from staff working in these 20 pediatric practices across Florida. Ware’s seven-point scale assessed satisfaction and burnout was measured using the six-point Maslach scale. The Medical Home Index measured the practice’s medical home characteristics. Descriptive and multivariate analyses were conducted. In total, 170 staff members completed the survey and the response rate was 42.6%.

**Results:**

Staff members reported high job satisfaction (mean 5.54; SD 1.26) and average burnout. Multivariate analyses suggest that care coordination is positively associated (b = 0.75) and community outreach is negatively associated (b = -0.18) with job satisfaction. Quality improvement and organizational capacity are positively associated with increased staff burnout (OR = 1.37, 5.89, respectively). Chronic condition and data management are associated with lower burnout (OR = 0.05 and 0.20, respectively). Across all models adaptive reserve, or the ability to make and sustain change, is associated with higher job satisfaction and lower staff burnout.

**Conclusions:**

Staff experiences in the transition to becoming a PCMH are important. Although our study is cross-sectional, it provides some insight about how medical home, staff and practice characteristics are associated with job satisfaction and burnout. Many PCMH initiatives include facilitation and it should assist staff on how to adapt to change. Unless staff needs are addressed a PCMH may be threatened by fatigue, burnout, and low morale.

**Electronic supplementary material:**

The online version of this article (doi:10.1186/2049-3258-72-36) contains supplementary material, which is available to authorized users.

## Background

Primary care in the United States is in transition. Shortcomings in primary care have been caused by workforce shortages [1, 2] as well as fragmented and expensive care. Child health and health outcomes are also subpar in the United States. For example, although youth obesity prevalence rates seem to have stabilized since 2003–2004, obesity prevalence continues to be high [3]. In addition, the Center for Disease Control and Prevention (CDC) finds [4] that the reported prevalence of ADHD, ASDs and bipolar disorders among children has increased during 1994 to 2011. A less than efficient pediatric primary health care system coupled with less than optimal outcomes points to much needed reform.

The movement to redesign primary care began in the 1960s. Numerous models and initiatives have been proposed over the past 50 plus years [5–7]. Specific to children, the medical home model was first proposed by the American Academy of Pediatrics (AAP) in 1967 [8]. A 2002 AAP position statement noted that care in the medical home should be, “accessible, continuous, comprehensive, family centered, coordinated, compassionate, and culturally competent [9]”. Other professional organizations, such as the AAP, American Academy of Family Physicians, American College of Physicians, and the American Osteopathic Association have also endorsed the medical home and published the Joint Principles of the Patient-Centered Medical Home [10].

Despite the length of time the medical home concept has been in existence, the widespread endorsement of the model, and support of the model in recent federal and state legislation such as the Affordable Care Act [11], evidence of its effectiveness is in the early stages [12]. For pediatrics, Homer’s 2008 systematic review found that none of the examined studies measured the medical home in its entirety and that the modest evidence that does exist is primarily focused on children with special health care needs [13]. Reasons for this paucity of evidence include lack of funding, difficulty in identifying and recruiting comparison practices, lack of evaluation standards, and lack of agreed upon tools. Some professional organizations have begun to address these challenges. The Commonwealth Fund created the Patient-Centered Medical Home Evaluators Collaborative in 2009 [14]. The objectives of this collaborative include reaching a consensus on a standard core set of outcome measures and instruments, sharing this consensus with interested researchers and facilitating an exchange of ideas and experiences among various medical-home evaluators. The collaborative has four workgroups: physician and staff experiences, cost and efficiency, patient experiences, and process and implementation metrics. Each group including the physician and staff experiences group represents an aspect of the medical-home evaluation design in which the collaborative seeks to achieve its objectives.

It is no surprise that physician and staff experiences are a key area of evaluation. Bohmer [15] observes that new primary care models such as the medical home need to be “proactive” (stresses the search and prevention of expected complications and comorbidities), “bundled” (focuses on long-duration episodes rather than isolated cases), and “shared” (involves care provided by a multi-disciplinary team). This means, according to Bohmer, in order to transition to such models organizational complexity must increase. This increase is a result of the need to increase practice size and professional diversity. The practice staff must deal with the change fatigue that comes with the necessary redefinition of their roles that such practice transformation entails [15, 16]. Therefore, it is important for the purpose of successful medical home design and evaluation to analyze how increasing the medical-home traits affects the staff. Studies that have previously investigated the association between staff and physician experiences and medical home characteristics include Lewis et al. [17] and Reid et al. [18]. Lewis and colleagues surveyed 382 clinical staff employed at 65 safety net clinics across five states. Results from the study suggest that an increased number of medical home characteristics were associated with better staff morale and lower levels of burnout. Reid and colleagues also studied experiences of staff employed at a medical home prototype clinic in Seattle. They found that staff at the medical home clinic had lower emotional exhaustion compared to staff at a comparison clinic. Although these studies suggest a positive association between medical home characteristics and staff morale, we believe our paper makes several contributions.

Our study focuses on staff at 20 pediatric practices participating in the Florida Pediatric Medical Home Demonstration Project. We use the data collected during the first year of the project to measure the differing levels of medical home characteristics already present in the practices, and how these have affected staff satisfaction and burnout across practices. To the best of our knowledge, our study is the first to analyze the effect of the medical-home transformation on staff morale and burnout of exclusively pediatric practices. Unlike the previous studies [17, 18], we use formal econometric analysis and a standardized instrument in the Medical Home Index (MHI) to investigate the effect of the medical home on staff satisfaction and burnout. Our hypothesis is that an increased number of medical home characteristics will be associated with lower levels of burnout and higher levels of job satisfaction.

## Methods

### Florida’s pediatric medical home demonstration project

Florida’s Pediatric Medical Home Demonstration project began in July 2011 with the recruitment, via email solicitation, of pediatric primary care practices from around the state. Practices could apply if they met three criteria: 1) a minimum of 100 children with special health care needs (CSHCN), 2) participation in Medicaid, a Medicaid health plan, or Florida KidCare, and 3) no former participation in another regional medical home implementation project. Applications were accepted until July 20, 2011. The applicants were scored on the basis of 24 items. Items included parent partner participation, number of publicly-insured CSHCN and the number of physicians in the practice supporting the project. The 20 top-scoring practices were selected for the project. Practices received no financial incentives to participate.

There are two parts to the project: 1) a facilitated quality improvement project led by the American Academy of Pediatrics [19] and 2) an independent, multi-stakeholder evaluation. The AAP project, which occurred during the first 16 months, included monthly conference calls, three face-to-face learning sessions, collection of monthly quality improvement data, and one-on-one mentorship by experts. The four-year evaluation includes components such as annual surveys with the core project team (three-person team including a lead physician), staff, and parents whose children receive care at the practices; on-site interviews; and, practice-level results of the Children’s Health Insurance Program Reauthorization Act core measures [20].

### Sample

**Figure 1:**
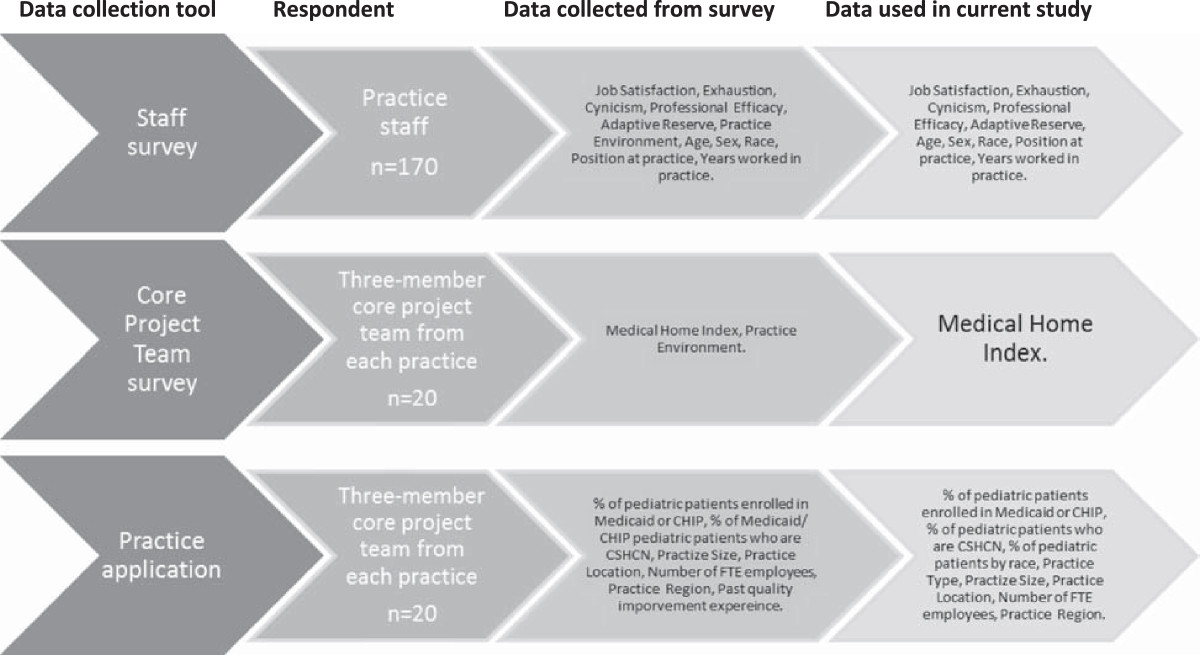
**Data used in the study and their sources.**

Baseline staff surveys are the primary data used in this cross sectional study. Between October and November 2011 staff at each of the 20 practices was given hard copy surveys to complete. All staff was asked to participate regardless of position. Staff was given a packet that included the survey, instructions for submission, and a return envelope. No incentives were given. It was requested that staff mail back the survey within 14 days. Reminders were sent via email at two, four, and six weeks. Flyers were posted in common areas of the practices to encourage staff participation. Overall, 170 surveys were completed (overall response rate 42.6%; by practice 13% to 100%).Data from the staff survey were matched to two supplemental datasets: 1) data from the practice’s original project application (which was completed and submitted by the core project team by 20 July 2011), and 2) data from the core project team survey completed between August and September 2011. Figure 1 describes the data sources. This study was approved by the Institutional Review Board at the University of Florida (#80-2011).

### Measures

Two outcome measures of professional experiences are assessed in this study: staff satisfaction and burnout. Satisfaction is measured using the overall job satisfaction score as devised by Warr et al. [21]. Staff was asked to rate their job satisfaction on a seven-point scale, with seven denoting the highest level of satisfaction. Burnout is assessed using the Maslach Burnout Inventory-General Scale, a six-point scale [22]. The scale has three domains: exhaustion, cynicism and professional efficacy. Higher burnout is indicated by greater scores for cynicism and exhaustion and a lower score for professional efficacy. Following guidance from Maslach et al. [22], the domain scores were categorized as low, average, or high. For the exhaustion domain, 0–2.00 denotes low, 2.01- 3.19 average, and 3.20-6.00 high. For cynicism, 0–1.00 is low, 1.01 to 2.19 average, and 2.20-6.00 high. For professional efficacy 0–4.00 is low (high burnout), 4.01 to 4.99 average, and 5.00-6.00 high (low burnout).

The primary independent variable of interest, medical home characteristics, is measured using the MHI [23]. The MHI includes six domains: Organizational Capacity, Chronic Condition Management, Care Coordination, Community Outreach, Data Management, and Quality Improvement. Domains are scored from 1 to 8, with higher scores indicating that the practice has more characteristics of a patient-centered medical home. The total MHI score ranges from 0–100 with higher scores denoting more medical home characteristics.

Finally, several staff and practice level characteristics are also included in the analyses. Staff characteristics include age, gender, years worked in the practice, race, position (clinical, administrative, or other), and adaptive reserve. Adaptive reserve, coined by Nutting and colleagues in their seminal TransforMED study of 36 family practices across the nation, is “an internal capability for organizational learning and development” [24]. Adaptive reserve is measured with the 23-item scale from the TransforMED Practice Environment Checklist [25]. Topics covered include willingness to change, problem solving, communication, and team dynamics. Items are scored using a five-point Likert scale. Total adaptive reserve was transformed to a 100 point scale where 100 indicates the highest level of adaptive reserve. Practice characteristics include percentage of pediatric patients enrolled in Medicaid or Florida’s Children’s Health Insurance Program (CHIP), percentage of pediatric patients enrolled in Medicaid or Florida’s CHIP with special healthcare needs, practice size, practice location, practice region, and number of full time equivalent employees.

### Analyses

Summary statistics are produced to describe the characteristics of the staff that completed the survey, practice characteristics, and medical home characteristics.

Multivariate regressions are performed to determine associations between staff satisfaction and medical home characteristics. Two regression models are estimated using ordinary least squares regression since the dependent variable is linear. The first ordinary least squares regression estimates the association between overall job satisfaction score and MHI total score. For the second ordinary least squares regression the six domains of the MHI are included as independent variables instead of MHI total score. Staff and practice characteristics are adjusted for in all models.

Multivariate regressions are also performed to determine associations between staff burnout and medical home characteristics. Six ordered logit regression models are used to estimate the association between the independent variables and the burnout domains. Ordered logit regressions are used given the functional form of the three burnout domains. Consistent with the literature [22], the domain scores are grouped into three ordinal categories of low, average, and high. Two ordered logit models are estimated for each of the three burnout domains. First, an ordered logit regression is estimated where the burnout domain was the dependent variable and MHI total score the independent variable of interest. Second, the same ordered logit regression is estimated, but the MHI domains are used instead of total MHI score. Staff and practice characteristics are adjusted for in all models.

To account for any unobserved effect common to staff working at the same practice, the standard errors estimated in all the models are controlled for by clustering at the practice level. The standard errors were calculated according to Liang and Zeger [26] that controls for clustering at the practice level. This approach is consistently used in other PCMH evaluations [17]. All analyses were conducted using Stata [27].

## Results

### Sample characteristics

Staff in the sample are mainly female (86.5%), White non-Hispanic (57.3%), and have been working in the practice for less than five years (63.2%) (Table 1). The staff seem to be equally distributed in age, and there is more clinical staff (69). Mean adaptive reserve score across all staff is 63.41 (standard deviation 17.81; range 4.35- 95.65).

**Table 1:** **Characteristics of the practice staff surveyed**

	Frequency	Percent
**Age**	**152**	
20-30 years	38	25.0%
31-40 years	36	23.7%
41-50 years	40	26.3%
50+ years	38	25.0%
**Gender**	**156**	
Female	135	86.5%
Male	21	13.5%
**Race/Ethnicity**	**150**	
White, non-Hispanic	86	57.3%
Black, non-Hispanic	21	14.0%
Hispanic	32	21.3%
Asian or pacific islander	7	4.7%
Other	4	2.7%
**Position at practice**	**149**	
Nurse-RN, LPN	24	16.1%
Social worker	1	0.7%
Clinician-PA, ARNP, MD, DO	44	29.5%
Administration-secretary, finance, fecords, office manager	43	28.9%
Other	37	24.8%
Clinical staff positions	69	46.3%
Non-clinical staff positions	80	53.7%
**Years worked in the practice**	**152**	
0-5 years	96	63.2%
6-10 years	24	15.8%
11-15 years	14	9.2%
16-20 years	11	7.2%
20+ years	7	4.6%
	**Mean**	**Standard deviation**
**Adaptive reserve score**	63.41	17.81

### Medical home and practice characteristics

**Figure 2:**
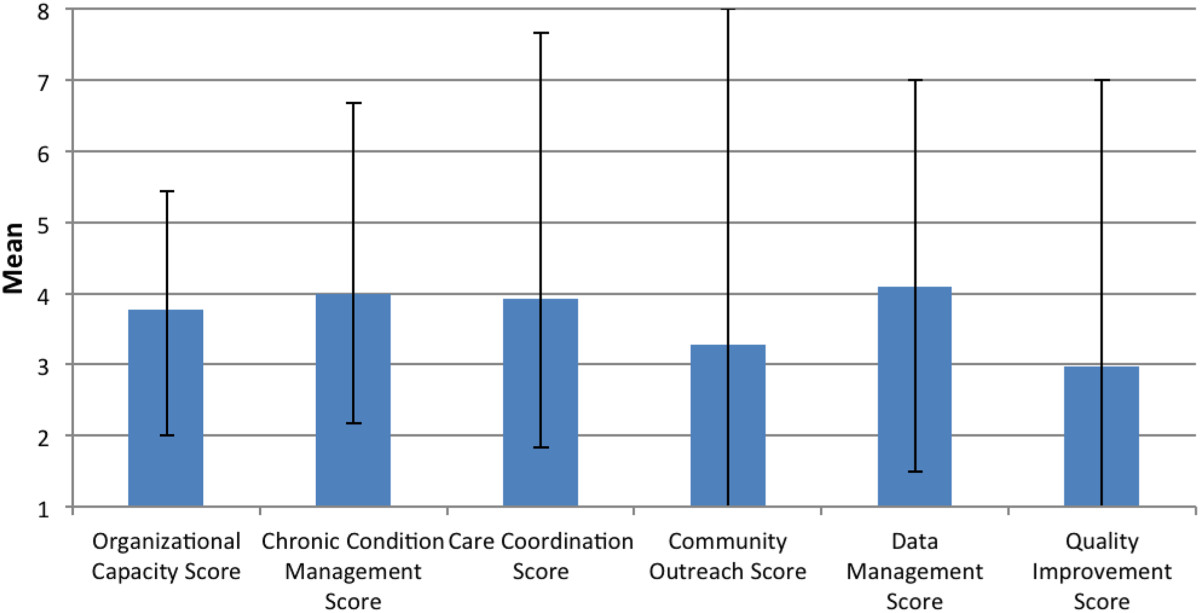
**Average baseline medical home index domain scores of the 20 practices.**

Figure 2 shows the average MHI domain scores for the 20 practices. Practices score highest in data management (4.10) and lowest in quality improvement (2.98). Although not shown in Figure 2, the baseline average total MHI score is 39.83 out of 100 (standard deviation (SD) 15.04), and across the practices, ranges from 14.29 to 80.

Table 2 shows that most of the practices in the sample are large practices and that most are in urban areas. Mean percentage of pediatric patients in the practices that are enrolled in Medicaid or Florida’s CHIP was 58.7%, and the mean percentage of those patients who have special healthcare needs is 31.3%. Mean number of full time equivalent employees in the practices is 15.

**Table 2:** **Characteristics of the practices participating in the Florida Pediatric Medical Home Demonstration Project**

	Frequency	Percent
**Size**	**20**	
Solo	5	25.0%
Small Practice (3 or fewer physicians)	6	30.0%
Large Practice (more than 3 physicians)	9	45.0%
**Location**	**20**	
Urban	13	65.0%
Suburban	7	35.0%
**Region**	**20**	
North Florida	5	25.0%
Central Florida	8	40.0%
South Florida	7	35.0%
	**Mean**	**Standard deviation**
**Percent of pediatric patients enrolled in Medicaid/CHIP**	58.7	22.3
**Percent of pediatric patients enrolled in Medicaid/CHIP that have special health care needs**	31.3	20.2
**Number of full-time equivalent employees**	15	13

Note: CHIP stands for Children’s Health Insurance Program.

### Outcome measures

Table 3 presents the summary statistics of the outcome variables. Across all staff, the average overall job satisfaction score is 5.54 (SD 1.26). Average scores are 2.31 for exhaustion (SD 1.49), 1.29 for cynicism (SD 1.30), and 5.03 for professional efficacy (SD 0.91).

**Table 3:** **Summary statistics of outcome variables**

Variable	Observations	Mean	Standard deviation	Minimum	Maximum	Possible maximum
Overall job satisfaction score	169	5.54	1.26	1.00	7.00	7.00
Exhaustion subscale	170	2.31	1.49	0.00	6.00	6.00
Cynicism subscale	170	1.29	1.30	0.00	5.20	6.00
Professional efficacy subscale	167	5.03	0.91	2.33	6.00	6.00

### Multivariate results

Table 4 presents the results of the ordinary least squares regressions where overall job satisfaction score is the dependent variable. Overall MHI was not associated with overall job satisfaction after controlling for covariates. Among covariates, adaptive reserve was significantly and positively associated with job satisfaction in both the model with overall MHI and the model with individual MHI components. In the second ordinary least squares regression, two individual components of MHI were associated with staff job satisfaction. A one-unit increase in care coordination score was associated with a 0.75 unit greater score in overall job satisfaction. Alternately, the level of community outreach of the practice had a negative association with job satisfaction. Anone-unity increase in community outreach was associated with a 0.18 unit decrease in job satisfaction. In this model two covariates were significantly associated with job satisfaction; practice size was significantly and positively associated with job satisfaction while a greater number of years working at the practice was negatively associated with job satisfaction.

**Table 4:** **Ordinary least squares regressions of overall job satisfaction on medical home, staff and practice characteristics**

	Overall job satisfaction	Overall job satisfaction
	**(1)**	**(2)**
MHI score	-0.01	
	(0.443)	
Organizational capacity		-0.20
		(0.259)
Chronic condition management		0.00
		(0.996)
Care coordination		0.75**
		(0.004)
Community outreach		-0.18**
		(0.030)
Data management		-0.17
		(0.086)
Quality improvement		0.00
		(0.893)
Adaptive reserve score	0.05**	0.05**
	(0.000)	(0.000)
Age		
31-40 years	-0.21	-0.25
	(0.573)	(0.540)
41-50 years	0.12	0.09
	(0.698)	(0.797)
50+ years	0.26	0.10
	(0.415)	(0.775)
Sex		
Male	-0.12	-0.07
	(0.572)	(0.716)
Race		
Black	-0.03	0.11
	(0.864)	(0.598)
Hispanic	0.05	0.17
	(0.838)	(0.509)
Asian/Pacific islander	-0.02	-0.06
	(0.960)	(0.881)
Other race category	-0.07	0.15
	(0.879)	(0.752)
Clinic position		
Administration	-0.12	-0.11
	(0.445)	(0.502)
Other job position	-0.05	-0.02
	(0.812)	(0.939)
Years worked in practice		
6-10	0.06	-0.01
	(0.761)	(0.965)
11-15	-0.14	-0.19
	(0.591)	(0.405)
16-20	-0.27	-0.29
	(0.491)	(0.405)
20+	-0.72	-0.79**
	(0.055)	(0.047)
% enrolled in Medicaid/CHIP	0.00	0.00
	(0.279)	(0.286)
% enrolled in Medicaid/CHIP w/SHCN	0.01	-0.01
	(0.237)	(0.069)
Practice size		
Small practice (3 or fewer)	-0.01	0.58**
	(0.952)	(0.027)
Large practice (more than 3)	-0.05	0.59**
	(0.952)	(0.042)
Practice location		
Suburban	0.12	0.10
	(0.502)	(0.518)
Number of full time employees	0.01	0.00
	(0.444)	(0.564)
Practice region		
North Florida	0.16	0.29
	(0.205)	(0.137)
South Florida	0.18	0.08
	(0.267)	(0.697)
Intercept	1.88**	1.67**
	(0.001)	(0.025)
R-squared	0.59	0.61
Number of observations	145	145

Note: ** = significant at the 5% level.

Referent groups are: 20–30 years old, female, White non-Hispanic, clinical staff, 0–5 years experience, solo practice, urban, and central Florida.

CHIP stands for Children’s Health Insurance Program, SHCN stands for special health care needs.

The results of the six ordered logit models predicting the three burnout domains are displayed in Table 5. Overall MHI was associated with increased odds of exhaustion, cynicism, and professional efficacy (Odds Ratio (OR) = 1.06, 1.11, and 1.09, respectively). In regard to the medical home characteristics, higher scores on the quality improvement domain are associated with increased odds of exhaustion, and higher scores on the chronic condition management component are associated with lower exhaustion (OR of 1.37 and 0.49, respectively). Organizational capacity is associated with increased odds (OR = 5.89), whereas data management is associated with decreased odds, of cynicism (OR = 0.20). Finally, higher organizational capacity and data management scores are associated with increased odds of professional efficacy (OR = 4.92 and 2.93, respectively).

**Table 5:** **Ordered logit regressions of burnout subscales on medical home, staff and practice characteristics**

Odds ratio for subscales						
	Exhaustion	Exhaustion	Cynicism	Cynicism	Professional efficacy	Professional efficacy
	(1)	(2)	(3)	(4)	(5)	(6)
MHI score	1.06**		1.11**		1.09**	
	(0.009)		(0.014)		(0.004)	
Organizational capacity		1.63		5.89**		4.92**
		(0.265)		(0.007)		(0.042)
Chronic condition management		0.49**		0.65		1.26
		(0.049)		(0.592)		(0.635)
Care coordination		0.54		0.43		0.23
		(0.402)		(0.649)		(0.151)
Community outreach		1.59		1.75		1.28
		(0.055)		(0.064)		(0.360)
Data management		1.08		0.20**		2.93**
		(0.786)		(0.021)		(0.009)
Quality improvement		1.37***		1.19		0.69
		(0.000)		(0.472)		(0.088)
Adaptive reserve score	0.95**	0.95**	0.88**	0.88**	1.08**	1.08**
	(0.000)	(0.000)	(0.000)	(0.000)	(0.000)	(0.000)
Age						
31-40 years	1.11	0.94	1.24	0.87	3.27**	6.83**
	(0.870)	(0.926)	(0.661)	(0.796)	(0.040)	(0.0017)
4150 years	1.09	1.15	0.62	0.65	5.12**	7.30**
	(0.921)	(0.871)	(0.385)	(0.481)	(0.048)	(0.024)
50+ years	0.71	0.88	0.16**	0.16	0.92	1.25
	(0.662)	(0.870)	(0.040)	(0.102)	(0.879)	(0.714)
Sex						
Male	0.68	0.60	3.24**	2.74	1.33	1.18
	(0.223)	(0.056)	(0.050)	(0.058)	(0.709)	(0.83)
Race						
Black	1.68	1.23	0.17**	0.21**	3.11	3.54
	(0.457)	(0.795)	(0.015)	(0.048)	(0.185)	(0.133)
Hispanic	0.78	0.88	0.91	0.89	1.86	1.74
	(0.566)	(0.793)	(0.892)	(0.918)	(0.363)	(0.404)
Asian/Pacific islander	1.41	1.78	0.94	0.69	0.14**	0.17**
	(0.718)	(0.499)	(0.960)	(0.670)	(0.006)	(0.007)
Other race category	1.85	1.32	0.25	0.12	0.40	0.19
	(0.592)	(0.839)	(0.269)	(0.299)	(0.404)	(0.071)
Clinic position						
Administration	1.64	1.36	2.86	2.19	1.66	2.49
	(0.098)	(0.271)	(0.059)	(0.172)	(0.371)	(0.152)
Other position category	1.19	1.04	1.23	0.82	3.81	5.12
	(0.660)	(0.921)	(0.717)	(0.747)	(0.139)	(0.087)
Years worked in practice						
6-10	0.62	0.66	1.49	1.47	0.58	0.77
	(0.527)	(0.616)	(0.527)	(0.605)	(0.358)	(0.708)
11-15	0.73	0.81	0.93	0.917	0.20**	0.27
	(0.591)	(0.731)	(0.941)	(0.941)	(0.027)	(0.129)
16-20	2.02	2.63	4.99	3.58	1.12	1.46
	(0.343)	(0.143)	(0.0.081)	(0.363)	(0.856)	(0.612)
20+	1.38	1.79	0.75	1.06	50.52**	83.59**
	(0.747)	(0.519)	(0.817)	(0.964)	(0.008)	(0.007)
% enrolled in Medicaid/CHIP	0.95**	0.95**	0.96**	0.94**	1.03**	1.07**
	(0.000)	(0.000)	(0.010)	(0.015)	(0.024)	(0.011)
% enrolled in Medicaid/CHIP w/SHCN	0.99	1.03	0.95**	1.03	0.96**	1.00
	(0.62)	(0.255)	(0.025)	(0.714)	(0.028)	(0.911)
Practice size						
Small (3 or fewer)	0.76	0.28	3.15	2.12	8.20**	1.51
	(0.652)	(0.163)	(0.209)	(0.660)	(0.002)	(0.695)
Large (more than 3)	0.48	0.19	1.64	1.86	18.26**	3.77
	(0.116)	(0.074)	(0.520)	(0.716)	(0.001)	(0.102)
Practice location						
Suburban	0.86	1.06	0.37	0.61	1.22	0.67
	(0.661)	(0.867)	(0.053)	(0.412)	(0.682)	(0.362)
Number of full time employees	1.03	1.03	1.01	1.03	0.95	0.95**
	(0.140)	(0.273)	(0.822)	(0.690)	(0.077)	(0.040)
Practice region						
North	1.70	4.40**	1.81	15.15	4.05**	1.15
	(0.245)	(0.010)	(0.273)	(0.074)	(0.041)	(0.887)
South	1.06	1.16	2.27	0.19	4.65**	8.86**
	(0.909)	(0.868)	(0.193)	(0.277)	(0.043)	(0.029)
Number of observations	145	145	145	145	145	145

Note: ** = significant at the 5% level.

Referent groups are: 20–30 years old, White, non-Hispanic, clinical staff, 0–5 years experience, solo practice, urban, and central Florida.

CHIP stands for Children’s Health Insurance Program, SHCN stands for special health care needs.

Across all six models, adaptive reserve was significantly associated with all three burnout domains. Adaptive reserve was positively associated with decreased odds of exhaustion and cynicism and increased odds of professional efficacy. The percentage of patients enrolled in Medicaid and CHIP was consistently associated with the dependent variables in all of the models. Results from that variable suggested that percentage of Medicaid and CHIP patients was positively associated with increased odds of professional efficacy and decreased odds of exhaustion and cynicism.

## Discussion and conclusion

This is the first study that assesses professional experiences in a pediatric PCMH project, and examines whether medical home characteristic have a positive association with these experiences. Our study uses survey data from 20 Florida practices participating in a pediatric medical home demonstration project. We find that different medical home characteristics are differently associated with satisfaction and burnout. Some characteristics are associated positively while others are associated negatively. We also find that individual staff characteristics, particularly adaptive reserve, seem to have a stronger association with satisfaction and burnout than medical home characteristics. Results from our descriptive and multivariate analyses extend the pediatric medical home literature in several ways.

First, results from our multivariate models of satisfaction are novel. These results seem to suggest that our hypothesis that more medical home characteristics would be associated with greater satisfaction was not confirmed (e.g., the total MHI score was not significant). Yet we identified specific medical home characteristics that were associated with job satisfaction. Our results suggest a direct relationship between care coordination and job satisfaction. Turchi et al. [28] found that families of children with special health care needs with adequate care coordination had increased odds of family-provider partnership, family centered care, and overall satisfaction. Perhaps those positive outcomes are fulfilling for staff as well and lead to greater job satisfaction. This would be an interesting future direction of research. The negative relationship we found between community outreach and satisfaction also merit further investigation. Community outreach encompasses building linkages with entities such as schools, community resources, as well as specialty clinics and hospitals. Perhaps staff are uncomfortable or frustrated with these type of tasks or view them beyond the scope of their duties. For example, creating linkages with schools could be an overwhelming activity as children in the practice attend many different schools, contact information at the schools may change frequently, and reasons for connecting to the schools vary.

Second, our multivariate analyses where burnout was the dependent variable are also novel. Our hypothesis that more medical home characteristics would be associated with decreased burnout was not confirmed. In fact, we found that higher MHI scores are associated with increased odds of burnout. Our results are in conflict with the Lewis study although they did not use the MHI to measure medical home characteristics [17]. However, when the MHI is broken down into domains our findings do note that some factors are associated with staff burnout. Increased chronic care management scores are associated with lower exhaustion. The medical home demands a systematic approach to chronic care management. Tools that facilitate this approach include registries, referral tracking, and care plans [29]. Such tools may also decrease staff exhaustion. Greater organizational capacity is associated with higher levels of cynicism. MHI items included in the organizational capacity domain encompass the provision of family-centered care at all levels of the organization. Several studies note the difficulties in implementing family centered care [30, 31]. Finally, our results suggest that higher data management scores are positively associated with professional efficacy. Although data management does not necessarily equate to having an electronic health record system, our finding when taken together with the evidence that find that advanced data management systems are beneficial to healthcare quality and efficiency [32–34] adds to the literature that implies that generally advanced data management systems are beneficial in healthcare.

Third, staff in our practices reported a level of satisfaction (5.54 out of 7.00) that is comparable to other primary care studies using Warr’s satisfaction scale. For example, job satisfaction was 5.99 out of 7.00 for a sample of 676 general practitioners in the European Practice Assessment study [35]. Warr’s scale has also been used extensively outside of health care such as the original study of blue collar, male workers in the manufacturing industry that found a satisfaction score of 5.33 [21]. The Organization for Economic Cooperation and Development has studied job satisfaction extensively [36]. A study of over 7,000 workers found that job satisfaction is affected by pay, hours of work, future prospects, how difficult the job is, job content, and interpersonal relationships [36].

Fourth, staff burnout in our sample is average (exhaustion and cynicism) to low (professional efficacy) using the cutoff points as published by the instrument’s developer. It is difficult to draw comparisons to the existing evidence on burnout given that there are hundreds of studies that cover a vast array of settings, positions, and experiences. Much of the work has been conducted outside of the U.S. Soler et al. [37] surveyed 1,393 primary care physicians in Europe and found that about one-third had high levels of burnout. Snibbe and colleagues studied burnout in primary care physicians and clinical staff in a large Health Maintenance Organization in the U.S. Findings suggest that burnout in primary care physicians was slightly higher than the Maslach cutoff scores [38].

Finally, although not a goal of our study, our findings emphasize the strong association between an individual’s adaptive reserve and professional experiences. Higher adaptive reserve was associated with greater job satisfaction and lower burnout in all models. Nutting and colleagues previously demonstrated that greater adaptive reserve among staff seemed to facilitate practices making and sustaining changes during PCMH transformation [25]. In this cross-sectional study, we found that staff experiences were generally more positive when staff has higher adaptive reserve.

Limitations exist in all studies and ours is no exception. Our response rate was 42.6%. Although this response rate is comparable to other surveys of health care workers [39], we do not have any information about the non-responders. Second, the MHI scores we used in the analyses were from the core project team, not the staff themselves. Incongruence may exist between staff and project team medical home perceptions. Third, we used the MHI to assess medical home characteristics. The MHI is unique in that it has been validated and used in several research studies. Yet, the MHI is based on self-assessment. We are not aware of any studies that comment on how well the MHI correlates with other tools such as the NCQA PCMH Recognition tool [40]. Fourth, our results may not be generalizable. The 20 practices of the study may not be representative of the pediatric primary care practices in Florida. As discussed earlier, the practices had to meet certain criteria such as participation in Medicaid, a Medicaid health plan, or Florida KidCare to be eligible to participate in the Florida Pediatric Medical Home Project. This may not be true for the majority of other Florida pediatric practices. Fifth, classification of staff into clinical and non-clinical was not straightforward. For example there was one social worker who was classified as non-clinical. It could be argued that a social worker is a clinical position, yet in doing so the results were unchanged. Finally, staff and physician experiences may change over time. Causation cannot be implied for our study. Longitudinal studies are needed to determine how professional experiences change throughout a transition.

Despite these limitations our results emphasize the importance of staff adaptability to change and how that can lead to improved outcomes. Introspection of this important issue will provide practice leaders with an idea of how easy or difficult the transformation process may be. Policymakers and health care organizations should ensure that medical home interventions assess and support staff as well as patients. Not only is this staff support critical, but it may help to mitigate turnover which is especially problematic in primary care [41, 42]. Interventions that focus on outcomes from a multitude of stakeholders, such as patient, staff, and health care systems, may be more sustainable in the long term.

## Authors’ original submitted files for images

Below are the links to the authors’ original submitted files for images. Authors’ original file for figure 1 Authors’ original file for figure 2
