# Supplementary figures and images for: Association between medical home characteristics and staff professional experiences in pediatric practices
Source: Arch Public Health. 2014 Oct 20;72:36. doi: 10.1186/2049-3258-72-36 (PMC4216343; doi:10.1186/2049-3258-72-36)

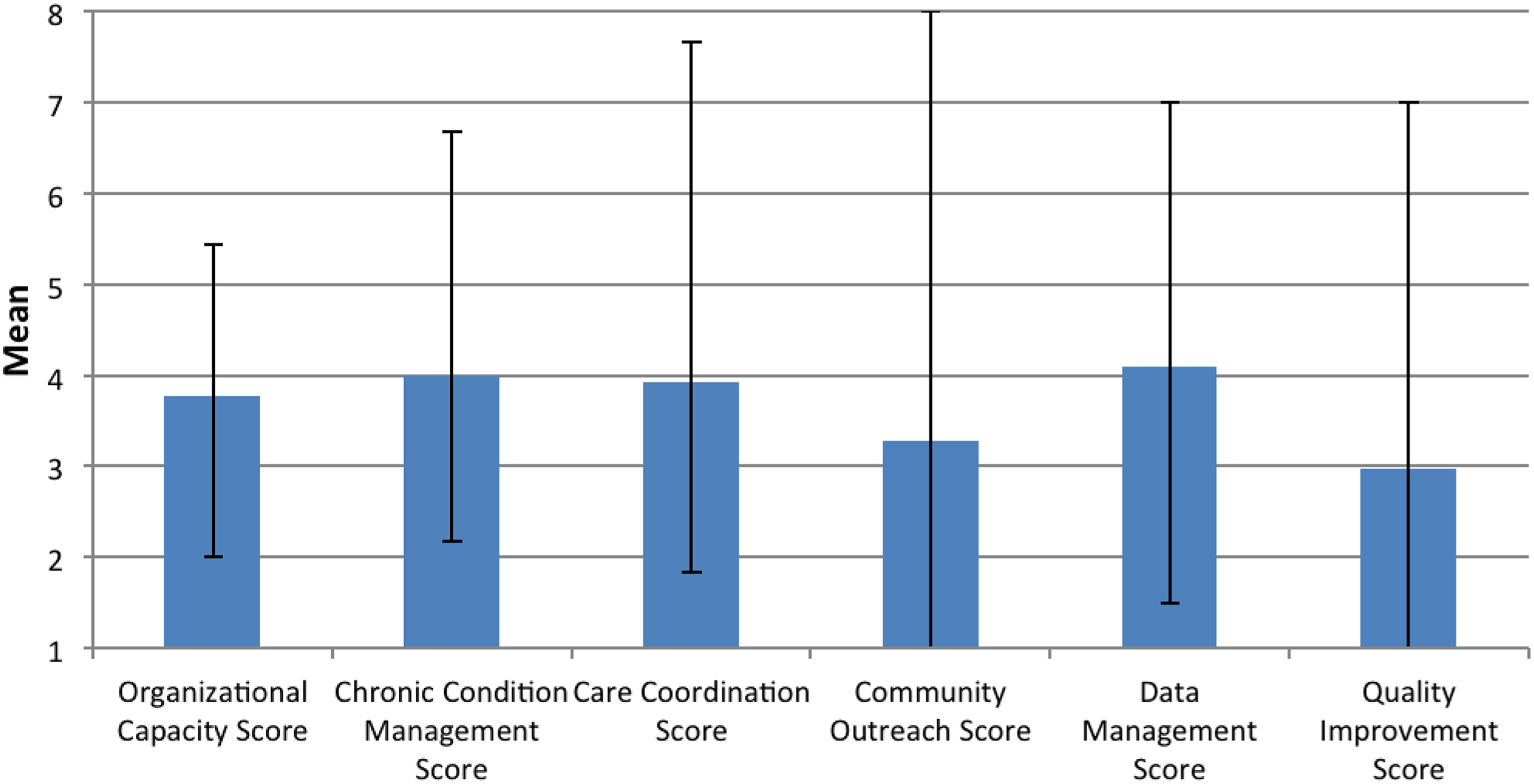

Supplement: Supplementary file 2 — Authors’ original file for figure 2 [file 13690_2014_5052_MOESM2_ESM.tif]
