# Supplementary material for: Association between medical home characteristics and staff professional experiences in pediatric practices
Source: Arch Public Health. 2014 Oct 20;72:36. doi: 10.1186/2049-3258-72-36 (PMC4216343; doi:10.1186/2049-3258-72-36)
Supplement: Supplementary file 1 — Authors’ original file for figure 1 [file 13690_2014_5052_MOESM1_ESM.pdf]

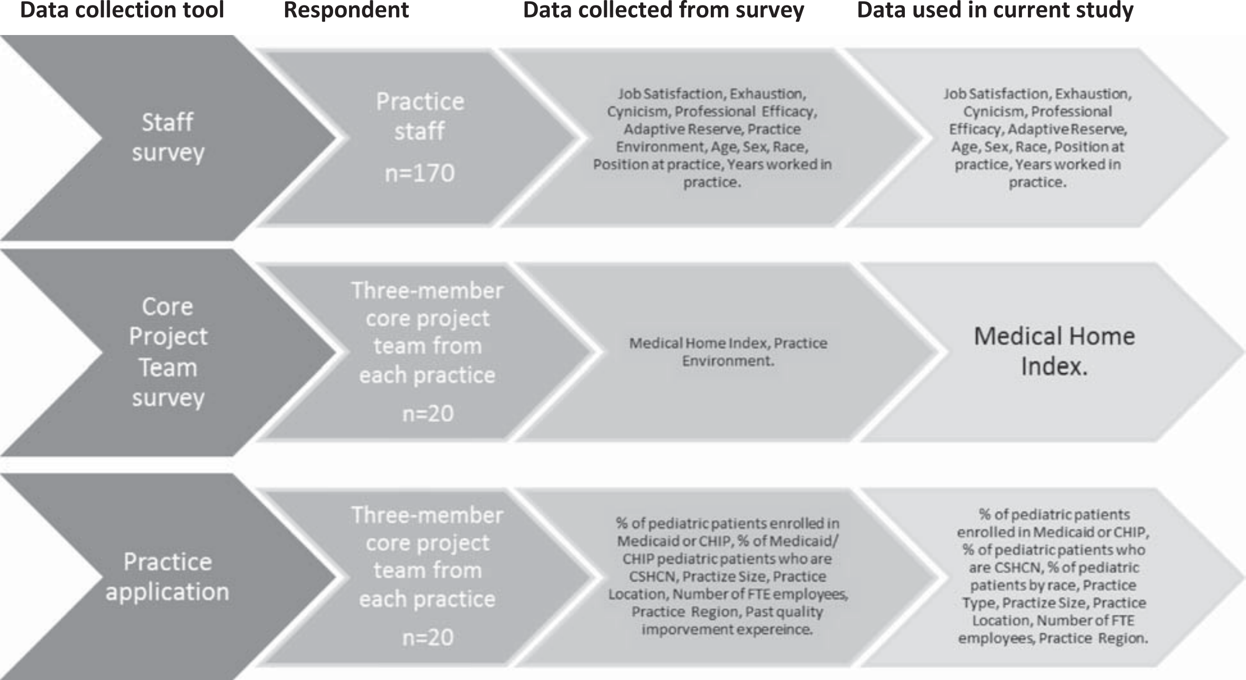
Data collection tool
Respondent
Data collected from survey
Data used in current study
Staff survey
Practice staff
n=170
Job Satisfaction, Exhaustion, Cynicism, Professional Efficacy, Adaptive Reserve, Practice Environment, Age, Sex, Race, Position at practice, Years worked in practice.
Job Satisfaction, Exhaustion, Cynicism, Professional Efficacy, Adaptive Reserve, Age, Sex, Race, Position at practice, Years worked in practice.
Core Project Team survey
Three-member core project team from each practice
n=20
Medical Home Index, Practice Environment.
Medical Home Index.
Practice application
Three-member core project team from each practice
n=20
% of pediatric patients enrolled in Medicaid or CHIP, % of Medicaid/ CHIP pediatric patients who are CSHCN, Practize Size, Practice Location, Number of FTE employees, Practice Region, Past quality imporvement expereince.
% of pediatric patients enrolled in Medicaid or CHIP, % of pediatric patients who are CSHCN, % of pediatric patients by race, Practice Type, Practize Size, Practice Location, Number of FTE employees, Practice Region.
